# Associations of maternal upper respiratory tract infection/influenza during early pregnancy with congenital heart disease in offspring: evidence from a case-control study and meta-analysis

**DOI:** 10.1186/s12872-019-1206-0

**Published:** 2019-12-02

**Authors:** Y. Q. Xia, K. N. Zhao, A. D. Zhao, J. Z. Zhu, H. F. Hong, Y. L. Wang, S. H. Li

**Affiliations:** 1 School of Public Health, Shanghai Jiao Tong University, 227 South Chongqing Road, Huangpu District, Shanghai, 200025 China; 2 China Hospital Development Institute, Shanghai Jiao Tong University, Shanghai, China; 3 Shanghai Children’s Medical Center, Shanghai Jiao Tong University School of Medicine, Shanghai, China; 4 Prenatal Diagnosis Department, International Peace Maternity & Child Health Hospital, Shanghai Jiao Tong University School of Medicine, 910 Hengshan Road, Xuhui District, Shanghai, 200030 China

**Keywords:** Early pregnancy, Upper respiratory tract infection, Influenza, Congenital heart disease

## Abstract

**Background:**

Evidences regarding the associations between maternal upper respiratory tract infection/influenza during pregnancy and the risk of congenital heart disease (CHD) is still controversial. This study was specifically designed to examine the associations by a case-control study and a meta-analysis of the published evidences and our finding.

**Methods:**

A hospital-based case-control study involving 262 children with simple CHD and 262 children with complex CHD, along with 262 control children, was conducted through June, 2016 to December, 2017. All children were aged 0–2 years old. Furthermore, a meta-analysis based on both previously published studies and our case-control study was performed.

**Results:**

In the case-control study, after adjusting for possible confounders, maternal upper respiratory tract infection/influenza during early pregnancy was found to be related to an increased risk of CHD (OR = 3.40 and 95% CI: 2.05–5.62 for simple CHD; OR = 2.39 and 95% CI: 1.47–3.88 for complex CHD). After a meta-analysis, the adverse impact was still kept significant (OR = 1.47 and 95% CI: 1.28–1.67 for simple CHD; OR = 1.44 and 95% CI: 1.14–1.75 for complex CHD). The very similar associations were also observed among single type of CHD, herein, ventricular septal defects (VSD) and tetralogy of fallot (TOF) in the case-control study. In the subsequent meta-analysis, however, the significant association only existed in VSD.

**Conclusions:**

Although there is still conflicting in TOF, the results are overall consistent, which provide new enforced evidence that maternal upper respiratory tract infection/influenza during early pregnancy, in general, play an important role in the occurrence of CHD.

## Background

Congenital heart disease (CHD) has been the most prevalent birth defect [1], and it remains to be an important cause of neonatal and infant deaths [2, 3]. It was reported that the global incidence of CHD had increased nearly 15 times over the past approximately 60 years, from 0.6‰ in 1930–1934 to 9.1‰ in 1995 [1]. In China, the rising trend similarly existed, and the prevalence of CHD among newborns had reached up to 11.1‰ [4]. Therefore, CHD has become a severe public health challenge all around the world.

The exact cause of CHD is still unclear. Generally, only 10–15% of CHD could be explained by gene defects and chromosome abnormalities, and the majority should be caused by the interaction of genetic factors and environmental exposures [5]. Early pregnancy is a key period for the development of fetal cardiovascular system, especially 14–60 days of gestation is the vulnerable window for CHD [6]. Upon the perspective of prevention, it is critically important to explore and identify CHD-related risk factors, especially those factors that can be intervened.

Upper respiratory tract infection and influenza always outbreak seasonally, in which season pregnant women are susceptible to the infection [7–9]. Previous studies have explored the relationship between maternal upper respiratory tract infection during pregnancy and the risk of CHD [10–25]. A number of evidence seemed to support the association [11, 16, 19, 20, 22, 23], however, a few other studies did not establish the relationship [12, 13]. Similarly, findings on the association between influenza during pregnancy and CHD was also controversial [11, 13–15, 18–21, 24, 25]. Almost all these studies took all CHD types as a whole. If the role of maternal upper respiratory tract infection/influenza varies in different type of CHD, then the possible different CHD type constitution may, at least partly, explains the inconsistent finding.

The present study aimed at exploring the associations of maternal upper respiratory tract infection/influenza during early pregnancy with CHD through a case-control study and a further meta-analysis. We observed if the associations were different between simple and complex CHD. Meanwhile, we paid particular attention to ventricular septal defects (VSD) and tetralogy of fallot (TOF), the most prevalent type of simple and complex CHD, respectively.

## Methods

### Hospital-based case-control study

#### Subjects

A hospital-based case-control study was conducted in Shanghai Children’s Medical Center through June, 2016 to December, 2017. Detailed study methods have been published elsewhere [26]. Briefly, study sample consisted of 524 cases (262 children with simple CHD and 262 children with complex CHD) and 262 controls without any birth defects. Among the controls, 132 was enrolled from pediatric respiratory medicine, 91 from pediatric general surgery, and 39 from pediatric gastroenterology. All the children were younger than 2 years old, and were recruited during the same period.

CHD was defined based on clinical diagnosis and verification by ultrasound. According to the codes of the International Classification of Diseases, Tenth Revision, Clinical Modification, complex CHD refers to all types of CHD except for simple CHD. The main types of simple CHD include VSD, secundum atrial septal defects, coarctation of aorta, patent ductus arteriosus, pulmonary stenosis, aortic valve stenosis, pulmonary stenosis, and etc. The main types of complex CHD include TOF, transposition of great arteries, double outlet right ventricle, hypoplastic left heart syndrome, tricuspid atresia and stenosis, and etc. [27, 28].

#### Ethical approval

The study conforms to the declaration of Helsinki, and the ethical application and consent procedure were approved by the Ethics Committee of Shanghai Jiao Tong University School of Medicine (Approval number: SJUPN-201717). Written informed consent was received from all participants prior to inclusion.

#### Associated factors of CHD

Information on sociodemographic characteristics and parental health-related behaviors and characteristics was retrospectively collected through the Parental Behaviors and Environmental Exposure Questionnaire (PBEQ), which has been used in our previous study [26]. The women who signed informed consent were invited to participate in an interview, and to fill in the PBEQ. Maternal upper respiratory tract infection/influenza exposure during early pregnancy was evaluated by two questions in the PBEQ. One is to ask: “Did you ever have upper respiratory infection (cold) and/or influenza during pregnancy?”, and the response was “yes” or “no”. Another one is a multiple-choice question regarding the period: “In which period of gestation did you get upper respiratory infection (cold) and/or influenza? The response is shown as: 1 for early pregnancy, 2 for second trimester, 3 for the third trimester.

##### Demographic and obstetric characteristics

There are 9 variables included parental ethnic, maternal age at delivery, maternal educational level, marital status, maternal residence, maternal prepregnancy obesity, maternal multiple births, infant gender and family history. The specific classification method of variables have previously been published [26].

##### Maternal health indicators during pregnancy

Maternal prepregnancy diabetes/hypertension (no/yes); maternal folic acid use (no/yes); and maternal smoking/drinking (defined as maternal previous history of smoking and/or drinking; no/yes).

#### Statistical analyses

Statistical descriptions including the number and percentage for categorical variables, and Chi-squared test was used to compare group difference. Logistic regression analyses were further applied to examine the crude and adjusted associations of maternal upper respiratory tract infection/influenza during early pregnancy with CHD. A two-step procedure was implemented for adjustments. Maternal ethic, maternal age at delivery, maternal education, marital status, residence, maternal prepregnancy obesity, multiple births, infant gender, and family history of CHD were adjusted in model Ι. In model II, prepregnancy diabetes/hypertension, folic acid use, smoking/drinking, were simultaneously further controlled. All the analyses were also repeated after controlling for the potential selection bias between cases and controls through propensity score matching, in which propensity score was calculated based on all covariates mentioned above. Propensity score was estimated by multivariable logistic regression model, in which all potential confounding variables related to CHD were included. The greedy nearest neighbor matching propensity score algorithm was applied to propensity score matching. Match the control with the case using caliper 0.1. R statistics software was utilized with Matchit software package.

All the analyses were performed with the Statistical Package for the Social Sciences (SPSS) (IBM-SPSS Statistics v22.0, Inc. Chicago, IL). A statistical significance level was set at a 2-tail *p*-value < 0.05.

### Meta-analysis

#### Literature search

We, furthermore, conducted a meta-analysis of all previous published studies and our case-control study on the associations between maternal upper respiratory tract infection/influenza and CHD. The relevant studies published up to Nov 4, 2018 were retrieved in the Pubmed, Embase, and Cochrane, without any language restriction, using the following terms: (‘pregnancy’ or ‘pregnant women’ or ‘mothers’ or ‘gravidity’ or ‘gestation’) and (‘heart defects, congenital’ or ‘congenital heart defects’ or ‘congenital heart disease’ or ‘heart abnormalities’ or ‘abnormalities, heart’ or ‘heart, malformation of’) and (‘respiratory tract infections’ or ‘infection, respiratory tract’ or ‘upper respiratory tract infections’ or ‘respiratory infection’ or ‘infections, respiratory’ or ‘common cold’ or ‘cold, common’ or ‘acute coryza’ or ‘coryza, acute’ or ‘influenza, human’ or ‘influenza’ or ‘flu’ or ‘grippe’) (the search strategy in Embase is shown in Additional file 1). Bibliographies of retrieved articles were also reviewed to identify additional eligible articles.

#### Inclusion and exclusion criteria

The retrieved literatures were included in the meta-analysis when they met all the following inclusion criteria: 1) Observational studies without any intervention that focused on pregnant women; 2) Outcome: The diagnosis of CHD in offspring was clearly presented and the diagnostic approach of cases and control groups was identical; 3) Exposure: Risk factors for CHD included maternal upper respiratory tract infection and/or influenza exposure during pregnancy; 4) Data: The information of odd ratio (OR) and 95% confidence interval (95% CI) could be obtained. As for the studies from the same researchers, the one with larger sample size would be included since the precision was driven primarily by the sample size.

#### Data extraction

For each included study, we extracted information as follows: name of author, publication year, country of origin, study type, sample size, type of CHD, maternal exposure including upper respiratory tract infection and influenza, and ORs (95% CIs). All data from original studies were extracted independently by two authors and compared afterwards. In cases of conflicting evaluations, any discrepancy would be resolved by a third reviewer.

#### Statistical analyses

ORs, along with corresponding 95% CIs, were applied to assess the associations of maternal upper respiratory tract infection/influenza during early pregnancy with CHD in offspring. Heterogeneity was determined with Cochran’s Q-statistic (significance level was set at *p* < 0.1), and was quantified with I^2^ test, in which I^2^>50% could be regarded as the moderate-to-high heterogeneity [29]. Random-effect model (DerSimonian-Laird method) was used if heterogeneity between studies was present; otherwise, a fixed-effect model (Mantel-Haenszel method) should be applied [30]. Additionally, the impact of individual studies on our meta-analysis summary statistic was evaluated by sensitivity analysis, which removes each study. In order to make our analysis more reliable, we compared the reappraised results with original results. Finally, publication bias was assessed by Begg’s test and Egger’s linear regression test, and the effect on the interpretation of results was estimated by the trim-and-fill computation [31]. All the statistical analyses were conducted using STATA version 14.0 (Stata Corp, College Station, TX, USA).

## Results

### Hospital-based case-control study

Among 262 children with simple CHD in this study, VSD was the most prevalent form (63.4%), and then atrial septal defects (51.9%) and patent ductus arteriosus (14.9%). For 262 children with complex CHD, TOF accounted the most (37.0%), and then transposition of great arteries (10.7%) and single ventricle (6.9%).

The sample characteristic was presented in Table 1. It could be seen that maternal folic acid use, maternal residence and maternal education level were significantly different between almost all CHD groups vs. Controls (all *p* < 0.05). In addition, infant gender and family history of CHD were also different, however, mainly between simple CHD and control (both *p* < 0.05), and the difference of smoking/drinking only existed between TOF and controls (*p* < 0.05). After propensity score matching, all these covariates were well balanced (all *p* > 0.05) (Additional file 2: Table S1).

**Table 1:** The description of characteristics by CHD Cases vs. Controls (n, %)

	All CHD			Simple CHD			Complex CHD			VSD			TOF		
	Cases (n=524)	Controls (n=262)	*p* value	Cases (n=262)	Controls (n=262)	*p* value	Cases (n=262)	Controls (n=262)	*p* value	Cases (n=60)	Controls (n=262)	*p* value	Cases (n=97)	Controls (n=262)	*p* value
Demographic and obstetric characteristics															
Maternal ethnic			0.236			0.243			0.337			1.000			0.716
Han	501,95.6	255, 97.3		250, 95.4	255, 97.3		251, 95.8	255, 97.3		58, 96.7	255, 97.3		93, 95.9	255, 97.3	
Other	30,4.4	7, 2.7		12, 4.6	7, 2.7		11, 4.2	7, 2.7		2, 3.3	7, 2.7		4, 4.1	7, 2.7	
Maternal age at delivery			0.543			1.000			0.302			0.235			0.391
<35 years old	473,90.3	240, 91.6		240, 91.6	240, 91.6		233, 88.9	240, 91.6		52, 86.7	240, 91.6		86, 88.7	240, 91.6	
≥35 years old	51,9.7	22, 8.4		22, 8.4	22, 8.4		29, 11.1	22, 8.4		8, 13.3	22, 8.4		11, 11.3	22, 8.4	
Maternal education			**<0.001**			**0.014**			**<0.001**			0.113			**<0.001**
Middle school and below	184,35.1	58, 22.1		83, 31.7	58, 22.1		101, 38.5	58, 22.1		21, 35.0	58, 22.1		40, 41.2	58, 22.1	
High school	117,22.3	48, 18.3		55, 21.0	48, 18.3		62, 23.7	48, 18.3		9, 15.0	48, 18.3		24, 24.7	48, 18.3	
College and above	223,42.6	156, 59.5		124, 47.3	156, 59.5		99, 37.8	156, 59.5		30, 50.0	156, 59.5		33, 34.0	156, 59.5	
Marital status			0.613			1.000			0.279			0.588			0.785
Married	511,97.5	257, 98.1		258, 98.5	257, 98.1		253, 96.6	257, 98.1		60, 100.0	257, 98.0		94, 96.9	257, 98.1	
Unmarried/divorced/widowed	13,2.5	5, 1.9		4, 1.5	5, 1.9		9, 3.4	5, 1.9		0, 0	5, 1.9		3, 3.1	5, 1.9	
Residence			**<0.001**			**<0.001**			**<0.001**			**0.017**			**<0.001**
Urban	209,39.9	166, 63.4		110, 42.0	166, 63.4		99, 37.8	166, 63.4		28, 46.7	166, 63.4		37, 38.1	166, 63.4	
Suburban /rural	315,60.1	96, 36.6		152, 58.0	96, 36.6		163, 62.2	96, 36.6		32, 53.3	96, 36.6		60, 61.9	96, 36.6	
Maternal prepregnancy obesity			0.523			0.432			0.108			1.000			0.104
Yes	23,4.4	9, 3.4		6, 2.3	9, 3.4		17, 6.5	9, 3.4		2, 3.3	9, 3.4		8, 8.2	9, 3.4	
No	501,95.6	253, 96.6		256, 97.7	253, 96.6		245, 93.5	253, 96.6		58, 96.7	253, 96.6		89, 91.8	253, 96.6	
Multiple births			0.857			0.178			0.381			0.594			0.067
Yes	44,8.4	23, 8.8		15, 5.7	23, 8.8		29, 11.1	2, 8.8		4, 6.7	23, 8.8		15, 15.5	2, 8.8	
No	480,91.6	239, 91.2		247, 94.3	239, 91.2		233, 88.9	239, 91.2		56, 93.3	239, 91.2		82, 84.5	239, 91.2	
Infant gender			**0.014**			**<0.001**			0.470			0.281			0.885
Male	288,55.0	168, 64.1		128, 48.8	168, 64.1		160, 61.1	168, 64.1		34, 56.7	168, 64.1		63, 64.9	168, 64.1	
Female	236,45.0	94, 35.9		134, 51.1	94, 35.9		102, 38.9	94, 35.9		26, 43.3	94, 35.9		34, 35.1	94, 35.9	
Family history of CHD			**0.024**			**0.005**			0.218			0.054			0.292
Yes	31,5.9	6, 2.3		20, 7.6	6, 2.3		11, 4.2	6, 2.3		5, 8.3	6, 2.3		5, 5.2	6, 2.3	
No	493,94.1	256, 97.7		242, 92.4	256, 97.7		251, 95.8	256, 97.7		55, 91.7	256, 97.7		92, 94.8	256, 97.7	
Maternal health indicators and behaviors															
Diabetes/hypertension			0.488			0.179			0.793			0.430			0.716
Yes	10,1.9	7, 2.7		2, 0.8	7, 2.7		8, 3.1	7, 2.7		0, 0	7, 2.7		4, 4.1	7, 2.7	
No	514,98.1	255, 97.3		260, 99.2	255, 97.3		254, 96.9	255, 97.3		60, 100.0	255, 97.3		93, 95.9	255, 97.3	
Folic acid supplementation			**0.001**			**0.044**			**<0.001**			**0.017**			**0.007**
Yes	390,74.4	222, 84.7		204, 77.9	222, 84.7		186, 71.0	222, 84.7		43, 71.7	222, 84.7		70, 72.2	222, 84.7	
No	134,25.6	40, 15.3		58, 22.1	40, 15.3		76, 29.0	40, 15.3		17, 28.3	40, 15.3		27, 27.8	40, 15.3	
Smoking/drinking			0.305			0.883			0.065			0.688			**0.027**
Yes	65,12.4	26, 9.9		25, 9.5	26, 9.9		40, 15.3	26, 9.9		7, 11.7	26, 9.9		18, 18.6	26, 9.9	
No	459,87.6	236, 90.1		237, 90.5	236, 90.1		222, 84.7	236, 90.1		53, 88.3	236, 90.1		79, 81.4	236, 90.1	

Entries in boldface mean *p*<0.05

The associations of maternal upper respiratory tract infection/influenza during early pregnancy with CHD in offspring were shown in Table 2. It was found that maternal upper respiratory tract infection/influenza during early pregnancy was a significant risk factor for both simple and complex CHD in univariate regression models. Through a two-step controlling procedure in multivariate regression models, the significant findings still remained. In the final full models, after adjusting for both demographic and obstetric characteristics and maternal health indicators during pregnancy, maternal upper respiratory tract infection/influenza during early pregnancy, compared to controls, could increase the risk of simple and complex CHD by 2.43 (OR = 3.43, 95% CI: 2.12–5.54) and 1.43 times (OR = 2.43, 95% CI: 1.54–4.45), respectively. Particularly, we also observed the similar associations in VSD (OR = 3.99, 95% CI: 1.94–8.18) and TOF (OR = 2.93, 95% CI: 1.56–5.52). When the analyses were repeated after propensity score matching, similar results were obtained (Additional file 2: Table S2).

**Table 2:** Associations of maternal upper respiratory tract infection/ influenza during early pregnancy with congenital heart disease in offspring

		Upper respiratory tract infection/ influenza		
	n	Crude OR (95% CI)	Adjusted OR (95% CI) ^a^	Adjusted OR (95% CI) ^b^
All CHD	524	2.58 (1.71–3.90)^***^	2.58 (1.69–3.95)^***^	2.56 (1.67–3.92)^***^
Simple CHD	262	3.43 (2.12–5.54)^***^	3.40 (2.06–5.61)^***^	3.40 (2.05–5.62)^***^
VSD	60	4.35 (2.23–8.49)^***^	4.09 (2.01–8.32)^***^	3.99 (1.94–8.18)^***^
Complex CHD	262	2.43 (1.54–3.84)^***^	2.43 (1.50–3.94)^***^	2.39 (1.47–3.88)^***^
TOF	97	2.68 (1.51–4.76)^**^	2.82 (1.51–5.29)^**^	2.93 (1.56–5.52)^**^

^a^ Model Ι: adjusted for maternal ethic, maternal age at delivery, maternal education, marital status, residence, maternal prepregnancy obesity, multiple births, infant gender, and family history of CHD

^b^ Model II: based on Model Ι, further adjusted for prepregnancy diabetes/hypertension, folic acid use, and smoking/drinking

^*^p value < 0.05; ^**^p value < 0.01; ^***^p value < 0.001

### Meta-analysis

**Fig. 1:**
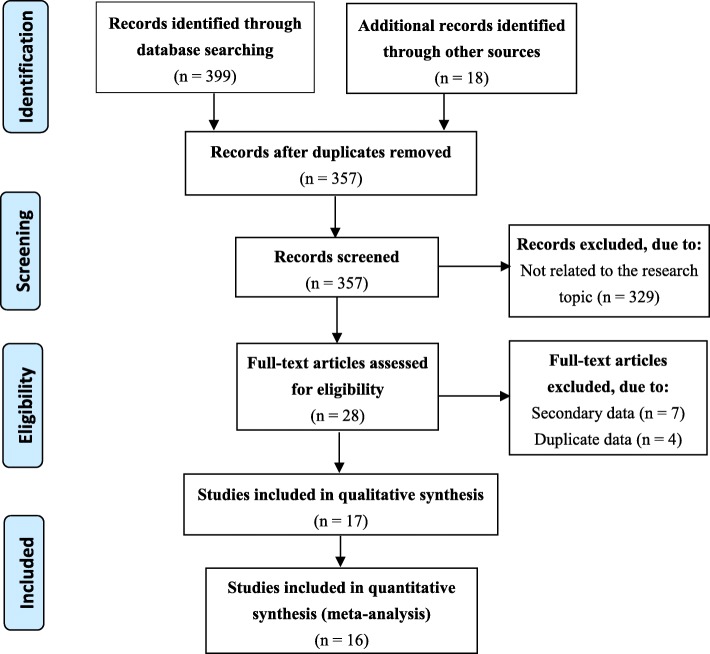
Flow diagram of the selection process of the included/excluded studies in the meta-analysis

The flow chart indicates the study process of literature selection and exclusion/inclusion criteria in the meta-analysis (Fig. 1). The 16 studies, including 11,911 cases and 74,358 controls, were finally identified as eligible in the analyses [10–25], covering a period from 1989 to 2018. All the 16 included studies were case–control studies. Among them, four only focused on maternal upper respiratory tract infection/influenza and the risk of CHD and the others included not only maternal upper respiratory tract infection/influenza but also other risks.

**Fig. 2:**
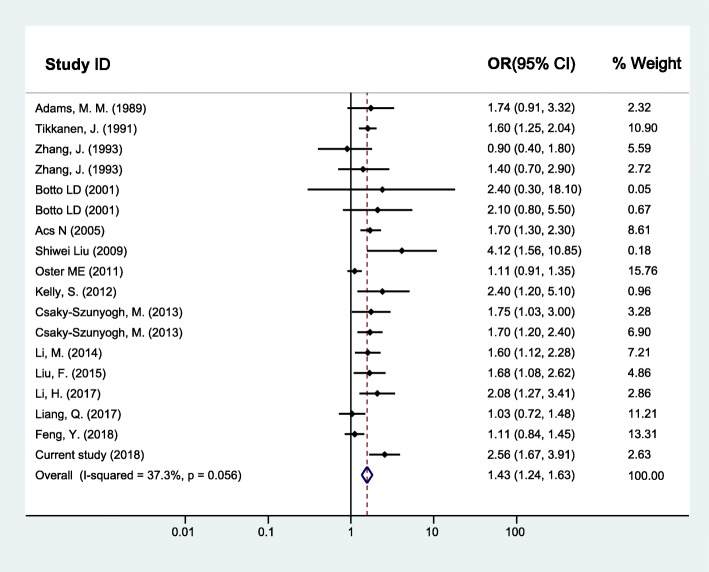
Forest plot of ORs and 95% CIs for the association of maternal upper respiratory tract infection/influenza and CHD

**Fig. 3:**
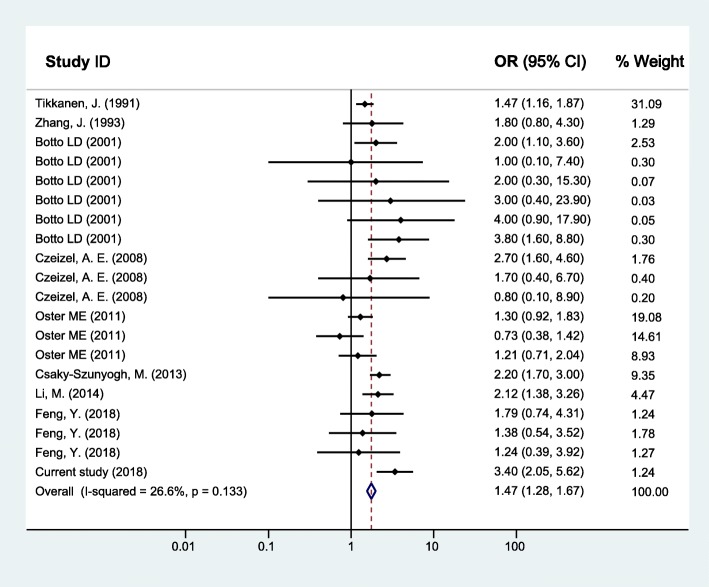
Forest plot of ORs and 95% CIs for the association of maternal upper respiratory tract infection/influenza and simple CHD

**Fig. 4:**
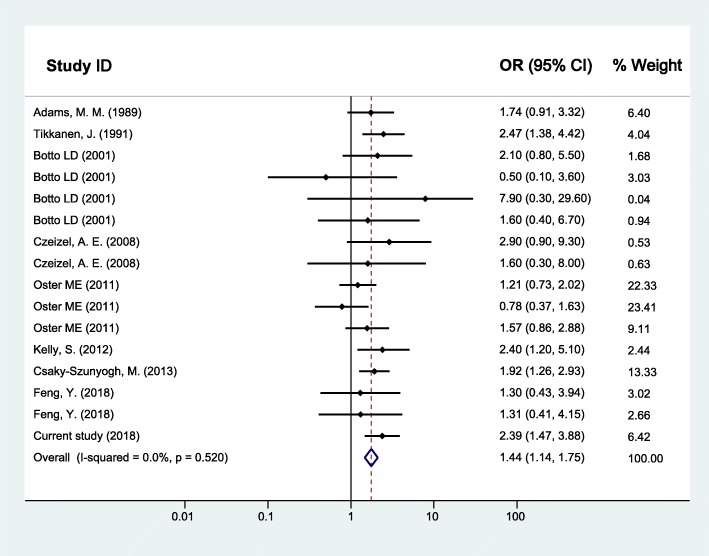
Forest plot of ORs and 95% CIs for the association of maternal upper respiratory tract infection/influenza and complex CHD

**Fig. 5:**
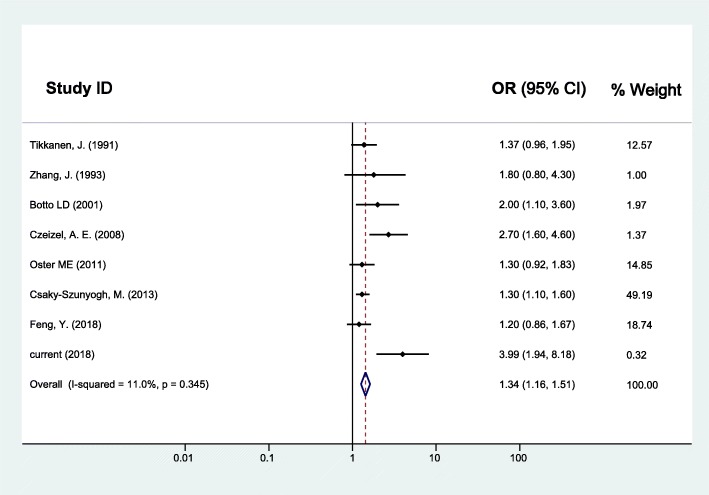
Forest plot of ORs and 95% CIs for the association of maternal upper respiratory tract infection/influenza and VSD

**Fig. 6:**
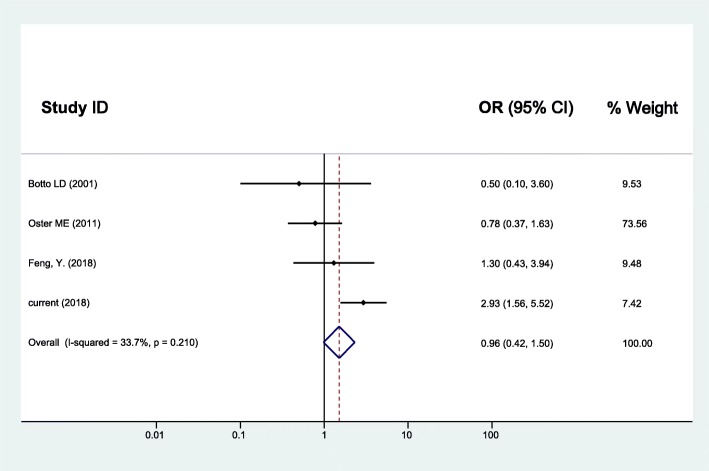
Forest plot of ORs and 95% CIs for the association of maternal upper respiratory tract infection/influenza and TOF

As for maternal upper respiratory tract infection/influenza and CHD, the random effects model was used since between-study heterogeneity existed (*I*^*2*^ = 37.3%, *p* = 0.056). The pooled analysis of the 16 studies, together with our study presented here, was performed and revealed significant associations with OR (95% CI) of 1.43 (1.24–1.63) (Fig. 2). Furthermore, the CHD was divided into two subgroups as simple CHD and complex CHD, and the two fixed effects models were applied since no significant between-study heterogeneity for simple CHD (*I*^*2*^ = 26.6%, *p* = 0.133) and complex CHD (*I*^*2*^ = 0.0%, *p* = 0.520) was found. Similarly, results of the pooled analyses showed that maternal upper respiratory tract infection/influenza during early pregnancy was associated with both simple CHD (OR = 1.47, 95% CI: 1.28–1.67) (Fig. 3) and complex CHD (OR = 1.44, 95% CI: 1.14–1.75) (Fig. 4). The comprehensive results showed that maternal upper respiratory tract infection/influenza had different effect sizes on VSD (OR = 1.34, 95% CI: 1.16–1.51) (Fig. 5) and TOF (OR = 0.96, 95% CI: 0.42–1.50) (Fig. 6). All the pooled analyses were also repeated based on our results after propensity score matching and the same 16 published studies, the associations were largely unchanged (Additional file 2: Figures S1-S5).

To examine the impact of individual study on the overall results, sensitivity analyses were carried out (Additional file 2: Figures S6-S10). The pooled ORs of maternal upper respiratory tract infection/influenza with CHD were not affected by the results of any individual study, suggesting the statistically robust results of this meta-analysis. Furthermore, publication bias was evaluated by performing Begg’s test and Egger’s linear regression test (statistical significance was set at *p* < 0.10). Neither Begg’s test nor Egger’s test found significant publication biases either in subgroup of simple CHD (z = 0.49, *p* = 0.626; t = 0.29, *p* = 0.776) or in complex CHD (z = 0.50, *p* = 0.620; t = − 0.33, *p* = 0.743). However, publication biases existed in all CHD as a whole group (t = 2.17, *p* = 0.046 in Egger’s test) (Additional file 2: Table S3). However, the further analysis with trim-and-fill test indicated that this level of publication bias did not impact the estimates (ie, no trimming done because data unchanged) [32].

## Discussion

Up to now, this is the largest population-based epidemiology study (*n* = 86,269) exploring the relationship between maternal upper respiratory tract infection/influenza and the risk of CHD in offspring. In this study, we examined the associations through a case-control study and a further meta-analysis based on previous evidences and our findings. Our examinations were performed not only in all CHD as a whole group but also in subgroups of CHD, simple CHD and complex CHD. Meanwhile, for the first time, we particularly examined the associations in VSD and TOF, the most prevalent single type of simple and complex CHD, respectively. The very consistent associations were established among different groups of CHD and even single types of CHD in our case-control study. In the subsequent meta-analysis, except for TOF, the similar consistent associations still existed. Generally, our study proposed enriched evidence that maternal upper respiratory tract infection/influenza during early pregnancy was implicated in CHD, although more studies are still needed to verify the association in each single type of CHD.

A number of previous studies have explored the impact of upper respiratory tract infection or influenza on CHD [11–17, 21–25]. There is considerable overlap in etiology and symptomatology between upper respiratory tract infection and influenza [33], and the symptoms of influenza are similar to the symptoms of upper respiratory tract infection, including fever, cough, sneezing, runny nose, sore throat, and etc. [33, 34]. For the common population and even medical professional, it is difficult to distinguish the two [34]. Therefore, we merged upper respiratory tract infection and influenza in the present study. A number of previous studies were conducted to explore the associations between upper respiratory tract infection or influenza and CHD [11–17, 21–25]. Usually, the studies observed the associations in CHD as a whole, without considering the possible difference among different type of CHD [11–17, 21–25]. However, although the information is still somewhat limited, the available evidence demonstrated that the impacts of upper respiratory tract infection/influenza varied among subtypes of CHD [10, 18–20]. In our hospital-based case-control study, we set up two case groups as simple CHD and complex CHD, respectively. To the best of our knowledge, this is the first study exploring the associations of maternal upper respiratory tract infection/influenza with simple CHD and complex CHD separately. Furthermore, we also observed the associations in VSD and TOF, the most prevalent single type of simple and complex CHD, respectively. Our case-control study showed that maternal upper respiratory tract infection/influenza could increase the risk of CHD, and the adverse impacts were consistent either in subgroups of CHD or single types of CHD. The subsequent meta-analysis, except for TOF, overally confirmed our findings, which verified our findings and further enforced the evidence between upper respiratory tract infection/ influenza and CHD.

In the present study, upper respiratory tract infection/ influenza was found to be a risk factor for TOF. As far as we know, to date only three previous studies specifically examine the associations, however, by contrast to our study, none of them got the similar findings [13, 17, 25]. The initial study to explore the correlation was a population-based case-control study conducted in Atlanta, the USA, in which 49 TOF cases vs. controls were analyzed, and among 49 cases, only one reported influenza exposure during pregnancy [13]. The limited case sample size may make the statistical model unstable and would affect the precision of the predictive value of the model. In addition, early pregnancy is the critical period for fetal cardiovascular development and, therefore, the susceptible window for CHD [6], however, the study collected information on influenza exposure without considering the stage of gestation. Similarly, another case-control study did not consider the susceptible period of influenza exposure during pregnancy for the risk of TOF [17]. A very recent study among 193 Chinese children with TOF examined the effects of influenza infection during different periods of pregnancy on the risk of TOF based on a 1:1 matched case-control study [25]. It seemed that maternal influenza exposure could increase the risk of TOF (OR ranged from 1.30 to 3.51 for influenza exposure during the first, second, and third trimester of pregnancy), although these results were not statistically significant. However, it is reasonable to deduce that the adverse effects would reach up to statistical significance with increasing sample size. Even so, it should be noticed that the value of OR in the second/third trimester was greater than that of the first trimester, which suggested that recalling bias should be a major limitation to be taken into account when explaining the results. Presently, it was difficult to clearly explain why our results were different from these previous studies. We hope more studies will be performed in the future to verify these results.

European surveillance of congenital anomalies (EUROCAT) study recorded a total prevalence of major congenital anomalies of 23.9 per 1000 births for 2003–2007, in which that CHD was the most common non-chromosomal subgroup, at 6.5 per 1000 births [35]. CHD is the most common cause of major congenital anomalies, forming a major global health burden [1]. In the present study, we detected maternal upper respiratory tract infection/influenza during first trimester perinatal period as an independent risk factor of CHD, even after a meta-analysis. Although the mechanism by which maternal upper respiratory tract infection/influenza may result in CHD is still unclear, several potential explanations are feasible in theory to support the relationship. The majority of upper respiratory tract infection is caused by viruses and bacteria, and influenza is always caused by viruses [36]. It was revealed that upper respiratory tract infection viruses such as respiratory syncytial virus could cause a cytokine related immunological or inflammatory response [37, 38]. Upper respiratory tract infection/influenza is often accompanied by fever, the mechanism may be same to which fever influence CHD. Previous studies have suggested that both fever [39, 40] and influenza virus infection [41–43] could induce the apoptotic death of cells. Apoptosis is known to be involved in cardiac morphogenesis, and altered apoptosis has been suggested to cause birth defect [40]. Another possible pathway is a direct effect of influenza virus inducing cell death [44].

Several limitations should be acknowledged in interpreting the results. First, although the diagnosis of CHD is more accurate in hospital-based case-control studies than in population-based case-control studies, selection bias cannot be inevitable. To reduce the potential selection bias between cases and controls, propensity score matching was used, however, the method is usually used in clinical intervention study. Secondly, our exposure data were collected by retrospective maternal self-reported information, thus recall bias is a concern. Thirdly, there was publication bias in CHD group, and it may have some effect on the outcome of meta-analysis.

## Conclusions

Although further studies are still needed to confirm the association of maternal upper respiratory tract infection/influenza with CHD, especially some single type of CHD, our findings, to a large extent, solve the present controversy and indicate that maternal upper respiratory tract infection/influenza during the first trimester of pregnancy should be an independent risk factor for CHD. As a result, pregnant women should exercise properly, strengthen the mother’s immunity to stay away from the source of infection.

## Supplementary information

**Additional file 1.** The search strategy in Embase.

**Additional file 2: Table S1.** The description of characteristics by CHD Cases vs. Controls (n, %) ----Propensity-score-matched. **Table S2.** Associations of maternal upper respiratory tract infection/ influenza during early pregnancy with congenital heart disease in offspring--- after a propensity-score-matched analysis. **Table S3.** The results of Begg’s test and Egger’s linear regression test for the association of maternal upper respiratory tract infection/influenza and CHD/simple CHD/complex CHD. **Figure S1.** Forest plot of ORs and 95% CIs for the association of maternal upper respiratory tract infection/influenza and CHD--- after a propensity-score-matched analysis. **Figure S2.** Forest plot of ORs and 95% CIs for the association of maternal upper respiratory tract infection/influenza and simple CHD--- after a propensity-score-matched analysis. **Figure S3.** Forest plot of ORs and 95% CIs for the association of maternal upper respiratory tract infection/influenza and complex CHD--- after a propensity-score-matched analysis. **Figure S4.** Forest plot of ORs and 95% CIs for the association of maternal upper respiratory tract infection/influenza and VSD--- after a propensity-score-matched analysis. **Figure S5.** Forest plot of ORs and 95% CIs for the association of maternal upper respiratory tract infection/influenza and TOF--- after a propensity-score-matched analysis. **Figure S6.** Plot of sensitivity analysis for the association of maternal upper respiratory tract infection/influenza and CHD. **Figure S7.** Plot of sensitivity analysis for the association of maternal upper respiratory tract infection/influenza and simple CHD. **Figure S8.** Plot of sensitivity analysis for the association of maternal upper respiratory tract infection/influenza and complex CHD. **Figure S9.** Plot of sensitivity analysis for the association of maternal upper respiratory tract infection/influenza and VSD. **Figure S10.** Plot of sensitivity analysis for the association of maternal upper respiratory tract infection/influenza and TOF.

## Data Availability

The database used for the study is not public but it is available from the corresponding author on reasonable request.

## Abbreviations

CHD: Congenital heart disease
PBEQ: The parental behaviors and environmental exposure questionnaire
SPSS: The statistical package for the social sciences
TOF: Tetralogy of fallot
VSD: Ventricular septal defects
