# Supplementary material for: Associations of maternal upper respiratory tract infection/influenza during early pregnancy with congenital heart disease in offspring: evidence from a case-control study and meta-analysis
Source: BMC Cardiovasc Disord. 2019 Dec 2;19:277. doi: 10.1186/s12872-019-1206-0 (PMC6889668; doi:10.1186/s12872-019-1206-0)
Supplement: Supplementary file 1 — Additional file 1. The search strategy in Embase. [file 12872_2019_1206_MOESM1_ESM.docx]

The used search strategy in Embase:

('congenital heart disease'/exp/mj OR 'congenital cardiac disease':ab,ti OR 'congenital cardiac distress':ab,ti OR 'congenital heart distress':ab,ti OR 'congenital heart failure':ab,ti OR 'heart congenital disease':ab,ti OR 'heart disease, congenital':ab,ti OR 'neonatal cardiopathy':ab,ti OR 'truncus arteriosus, persistent':ab,ti) AND ('pregnant woman'/exp/mj OR ' pregnancy'/exp/mj OR 'child bearing':ab,ti OR 'childbearing':ab,ti OR gestation:ab,ti OR gravidity:ab,ti OR 'intrauterine pregnancy':ab,ti OR 'labor presentation':ab,ti OR 'labour presentation':ab,ti OR 'pregnancy maintenance':ab,ti OR 'pregnancy trimesters':ab,ti) AND (('upper respiratory tract infection'/exp/mj OR 'upper respiration tract infection':ab,ti OR 'upper respiratory tract inflammation':ab,ti OR 'upper respiratory infection'/exp OR 'upper respiratory infection' OR 'infection, upper respiratory tract':ab,ti OR 'respiratory tract infection, upper':ab,ti OR 'tractus respiratorius superior infection':ab,ti OR 'upper airway infection':ab,ti) OR ('common cold'/exp/mj OR coryza:ab,ti OR 'natural cold':ab,ti OR 'cold, common':ab,ti OR 'common colds':ab,ti OR 'natural colds':ab,ti) OR ('influenza'/exp/mj OR 'bronchitis epidemica':ab,ti OR 'epidemic bronchitis':ab,ti OR 'bronchitis, epidemic':ab,ti OR flu:ab,ti OR flue:ab,ti OR 'influenza infection':ab,ti OR 'influenza syndrome':ab,ti OR 'influenza, human':ab,ti))
