# Supplementary material for: Associations of maternal upper respiratory tract infection/influenza during early pregnancy with congenital heart disease in offspring: evidence from a case-control study and meta-analysis
Source: BMC Cardiovasc Disord. 2019 Dec 2;19:277. doi: 10.1186/s12872-019-1206-0 (PMC6889668; doi:10.1186/s12872-019-1206-0)
Supplement: Supplementary file 2 — Additional file 2: Table S1. The description of characteristics by CHD Cases vs. Controls (n, %) ----Propensity-score-matched. Table S2. Associations of maternal upper respiratory tract infection/ influenza during early pregnancy with congenital heart disease in offspring--- after a propensity-score-matched analysis. Table S3. The results of Begg’s test and Egger’s linear regression test for the association of maternal upper respiratory tract infection/influenza and CHD/simple CHD/complex CHD. Figure S1. Forest plot of ORs and 95% CIs for the association of maternal upper respiratory tract infection/influenza and CHD--- after a propensity-score-matched analysis. Figure S2. Forest plot of ORs and 95% CIs for the association of maternal upper respiratory tract infection/influenza and simple CHD--- after a propensity-score-matched analysis. Figure S3. Forest plot of ORs and 95% CIs for the association of maternal upper respiratory tract infection/influenza and complex CHD--- after a propensity-score-matched analysis. Figure S4. Forest plot of ORs and 95% CIs for the association of maternal upper respiratory tract infection/influenza and VSD--- after a propensity-score-matched analysis. Figure S5. Forest plot of ORs and 95% CIs for the association of maternal upper respiratory tract infection/influenza and TOF--- after a propensity-score-matched analysis. Figure S6. Plot of sensitivity analysis for the association of maternal upper respiratory tract infection/influenza and CHD. Figure S7. Plot of sensitivity analysis for the association of maternal upper respiratory tract infection/influenza and simple CHD. Figure S8. Plot of sensitivity analysis for the association of maternal upper respiratory tract infection/influenza and complex CHD. Figure S9. Plot of sensitivity analysis for the association of maternal upper respiratory tract infection/influenza and VSD. Figure S10. Plot of sensitivity analysis for the association of maternal upper respiratory tract infection/infl [file 12872_2019_1206_MOESM2_ESM.pdf]

Table S1 The description of characteristics by CHD Cases vs. Controls (n, %) ----Propensity-score-matched

	**All CHD [a]**			**Simple CHD [a]**			**Complex CHD [a]**			**VSD [a]**			**TOF [a]**		
	**Cases (n=231)**	**Controls (n=231)**	***p* value**	**Cases (n=187)**	**Controls (n=187)**	***p* value**	**Cases (n=186)**	**Controls (n=186)**	***p* value**	**Cases (n=56)**	**Controls (n=98)**	***p* value**	**Cases (n=89)**	**Controls (n=149)**	***p* value**
**Demographic and obstetric characteristics**															
Maternal ethnic			0.360			1.000			0.557			0.971			0.926
Han	227, 98.3	224, 97.0		180, 96.3	180, 96.3		179, 96.2	181, 97.3		54, 96.4	93, 94.9		87, 97.8	144, 96.6	
Other	4, 1.7	7, 3.0		7, 3.7	7, 3.7		7, 3.8	5, 2.7		2, 3.6	5, 5.1		2, 2.2	5, 3.4	
Maternal age at delivery			0.873			1.000			0.375			0.776			0.945
<35 years old	209, 90.5	210, 90.9		172, 92.0	172, 92.0		171, 91.9	166, 89.2		50, 89.3	86, 87.8		81, 91.0	136, 91.3	
≥35 years old	22, 9.5	21, 9.1		15, 8.0	15, 8.0		15, 8.1	20, 10.8		6, 10.7	12, 12.2		8, 9.0	13, 8.7	
Maternal education			0.630			0.866			0.294			0.855			0.678
Middle school and below	67, 29.0	58, 25.1		48, 25.7	51, 27.3		65, 34.9	52, 28.0		19, 33.9	29, 29.6		33, 37.1	47, 31.5	
High school	44, 19.0	48, 20.8		44, 23.5	40, 21.4		33, 17.7	41, 22.0		8, 14.3	15, 15.3		23, 25.8	41, 27.5	
College and above	120, 51.9	125, 54.1		95, 50.8	96, 51.3		88, 47.3	93, 50.0		29, 51.8	54, 55.1		33, 37.1	61, 40.9	
Marital status			0.760			1.000			1.000			-			1.000
Married	225, 97.4	226, 97.8		184, 98.4	184, 98.4		182, 97.8	182, 97.8		56, 100.0	98, 100.0		86, 96.6	144, 96.6	
Unmarried/divorced/widowed	6, 2.6	5, 2.2		3, 1.6	3, 1.6		4, 2.2	4, 2.2		0, 0	0, 0		3, 3.4	5, 3.4	
Residence			0.261			0.408			0.604			0.715			0.362
Urban	123, 53.2	135, 58.4		87, 46.5	95, 50.8		86, 46.2	91, 48.9		28, 50.0	52, 53.1		37, 41.6	71, 47.7	
Suburban /rural	108, 46.8	96, 41.6		100, 53.2	92, 49.2		100, 53.8	95, 51.1		28, 50.0	46, 46.9		52, 58.4	78, 52.3	
Maternal prepregnancy obesity			0.805			0.760			0.359			0.962			0.829
Yes	8, 3.5	9, 3.9		6, 3.2	5, 2.7		4, 2.2	7, 3.8		2, 3.6	2, 2.0		6, 6.7	9, 6.0	
No	223, 96.5	222, 96.1		181, 96.8	182, 97.3		182, 97.8	179, 96.2		54, 96.4	96, 98.0		83, 93.3	140, 94.0	
Multiple births			0.517			0.673			0.293			0.926			0.425
Yes	19, 8.2	23, 10.0		13, 7.0	11, 5.9		15, 8.1	21, 11.3		3, 5.4	7, 7.1		14, 15.7	18, 12.1	
No	212, 91.8	208, 90.0		174, 93.0	176, 94.1		171, 91.9	165, 88.7		53, 94.6	91, 92.9		75, 84.3	131, 87.9	

Infant gender			0.101			0.836			0.085			0.692			0.404
Male	155, 67.1	138, 59.7		98, 52.4	100, 53.5		126, 67.7	110, 59.1		31, 55.4	51, 52.0		58, 65.2	89, 59.7	
Female	76, 32.9	93, 40.3		89, 47.6	87, 46.5		60, 32.3	76, 40.9		25, 44.6	47, 48.0		31, 34.8	60, 40.3	
Family history of CHD			0.313			0.500			0.760			0.656			1.000
Yes	3, 1.3	6, 2.6		3, 1.6	6, 3.2		5, 2.7	6, 3.2		4, 7.1	4, 4.1		3, 3.4	5, 3.4	
No	228, 98.7	225, 97.4		184, 98.4	181, 96.8		181, 97.3	180, 96.8		52, 92.9	94, 95.9		86, 96.6	144, 96.6	
**Maternal health indicators and behaviors**															
Diabetes/hypertension			1.000			1.000			1.000			-			0.925
Yes	5, 2.2	5, 2.2		2, 1.1	1, 0.5		4, 2.2	5, 2.7		56, 100.0	98, 100.0		4, 4.5	5, 3.4	
No	226, 97.8	226, 97.8		185, 98.9	186, 99.5		182, 97.8	181, 97.3		0, 0	0, 0		85, 95.5	144, 96.6	
Folic acid supplementation			0.708			0.518			0.453			0.611			0.668
Yes	194, 84.0	191, 82.7		147, 78.6	152, 81.3		142, 76.3	148, 79.6		42, 75.0	77, 78.6		69, 77.5	119, 79.9	
No	37, 16.0	40, 17.3		40, 21.4	35, 18.7		44, 23.7	38, 20.4		14, 25.0	21, 21.4		20, 22.5	30, 20.1	
Smoking/drinking			0.644			0.863			0.304			0.516			0.952
Yes	25, 10.8	22, 9.5		18, 9.6	19, 10.2		16, 8.6	22, 11.8		7, 12.5	9, 9.2		14, 15.7	23, 15.4	
No	206, 89.2	209, 90.5		169, 90.4	168, 89.8		170, 91.4	164, 88.2		49, 87.5	89, 90.8		75, 84.3	126, 84.6	

[a] Controls were matched to cases based on propensity-score-matched analysis.

Table S2 Associations of maternal upper respiratory tract infection/ influenza during early pregnancy with congenital heart disease in offspring ---after a propensity-score-matched analysis

	Upper respiratory tract infection/ influenza (Propensity-score-matched)		
	Crude OR (95% CI)	Adjusted OR (95% CI) [a]	Adjusted OR (95% CI) [b]
**All CHD**	**2.85（1.76-4.61）*****	**2.86（1.75-4.66）*****	**2.90（1.77-4.74）*****
**Simple CHD**	**3.47（2.00-6.01）*****	**3.55（2.03-6.23）*****	**3.56（2.03-6.25）*****
VSD	**3.08（1.40-6.80）****	**3.35（1.45-7.71）****	**3.40（1.46-7.90）****
**Complex CHD**	**2.28（1.33-3.92）****	**2.30（1.32-4.02）****	**2.23（1.27-3.91）****
TOF	**3.38（1.72-6.66）*****	**3.35（1.66-6.73）****	**3.39（1.67-6.86）****

[a] Model I: adjusted for maternal ethic, maternal age at delivery, maternal education, marital status, residence, maternal prepregnancy obesity, multiple births, infant gender, and family history of CHD.

[b] Model II: based on Model I, further adjusted for prepregnancy diabetes/hypertension, folic acid use, and smoking/drinking.

$^{*}p$ value <0.05; $^{**}p$ value <0.01; $^{***}p$ value <0.001

Table S3 The results of Begg's test and Egger's linear regression test for the association of maternal upper respiratory tract infection/influenza and CHD/simple CHD/complex CHD

**CHD**	**Begg test**	**Egger test**
CHD	z=1.36, p=0.173	t=2.17, p=0.046
Simple CHD	z=0.49, p=0.626	t=0.29, p=0.776
Complex CHD	z=0.50, p=0.620	t= - 0.33, p=0.743

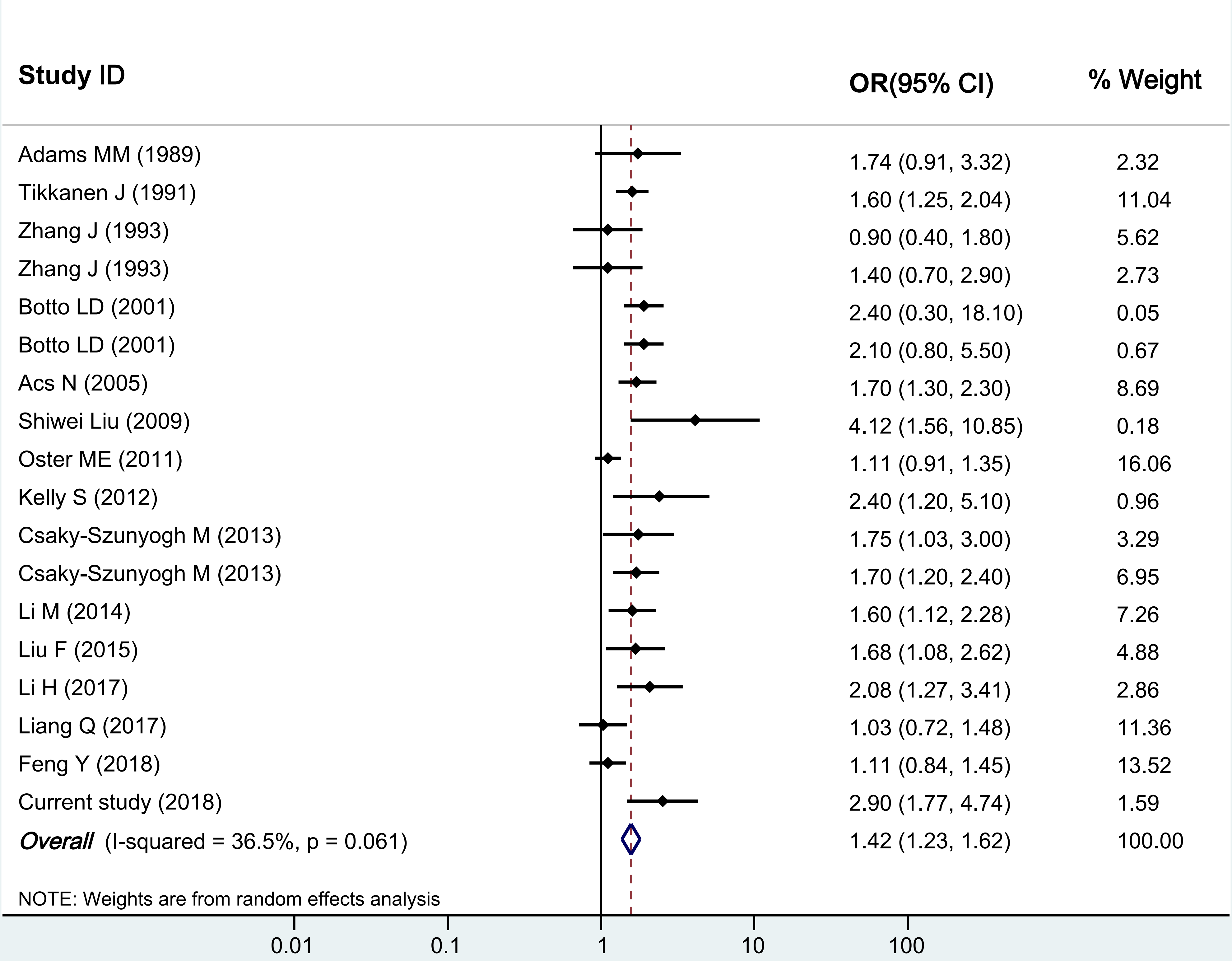

Figure S1 Forest plot of ORs and 95% CIs for the association of maternal upper respiratory tract infection/influenza and CHD--- after a propensity-score-matched analysis

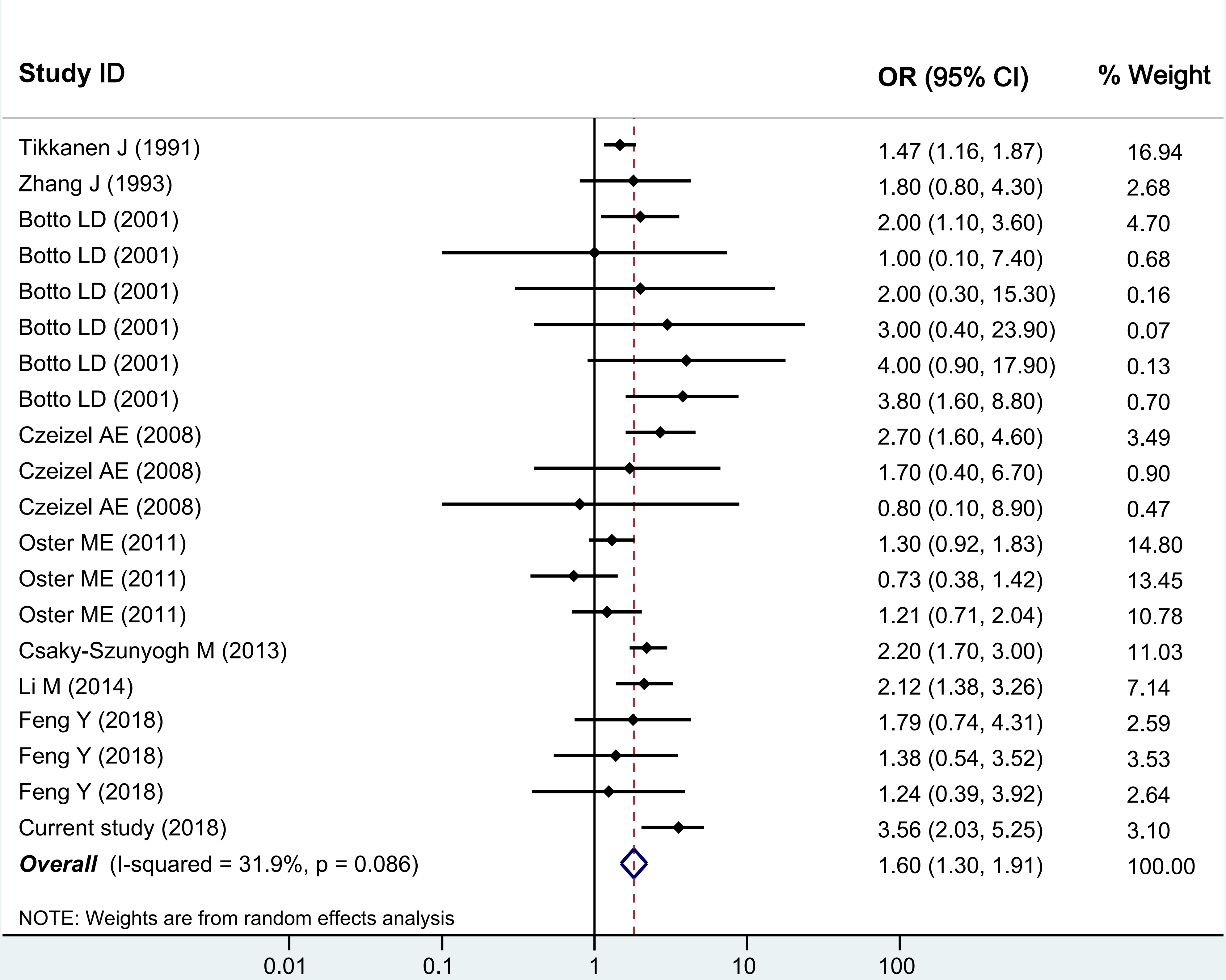

Figure S2 Forest plot of ORs and 95% CIs for the association of maternal upper respiratory tract infection/influenza and simple CHD--- after a propensity-score-matched analysis

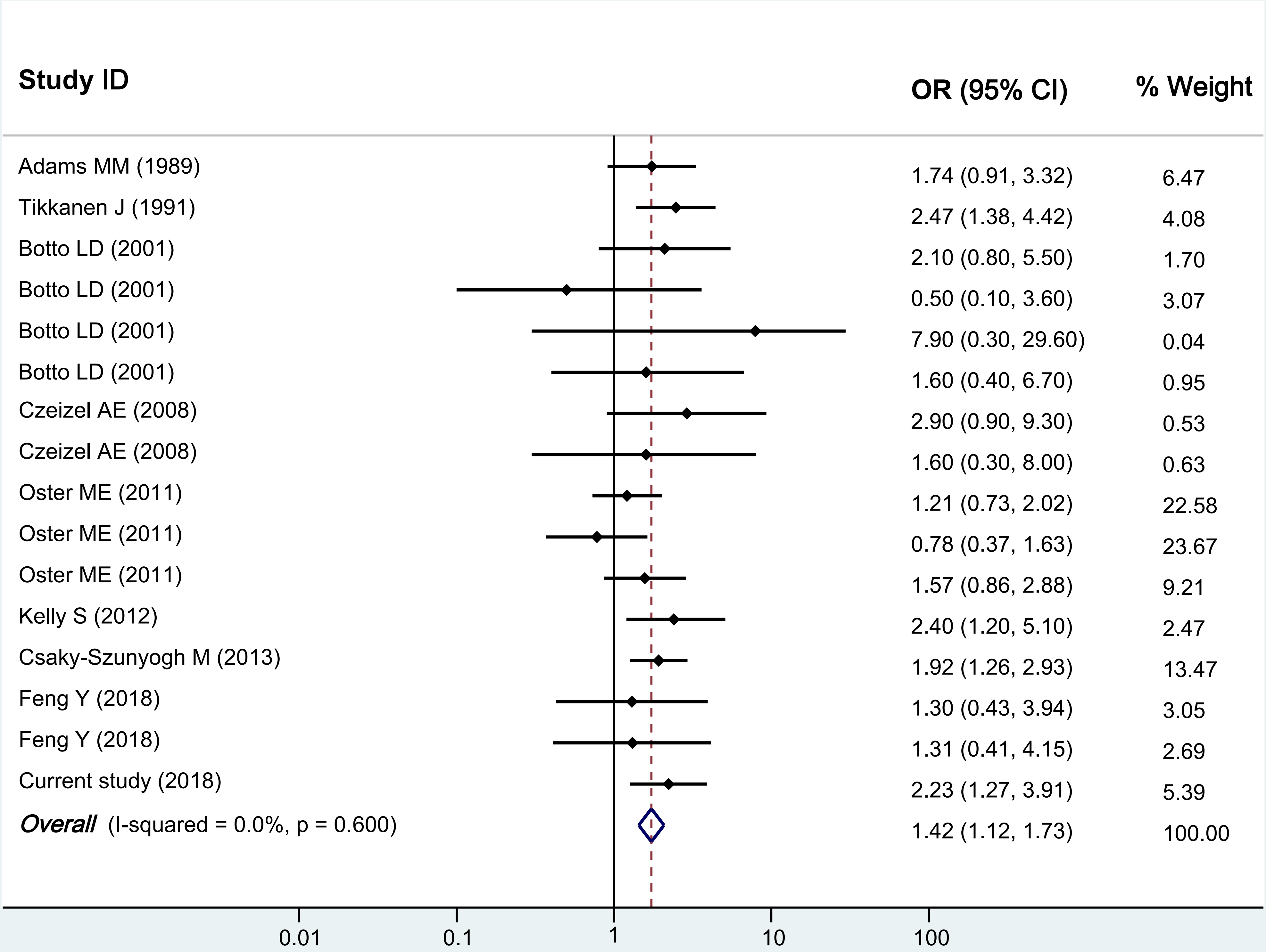

Figure S3 Forest plot of ORs and 95% CIs for the association of maternal upper respiratory tract infection/influenza and complex CHD--- after a propensity-score-matched analysis

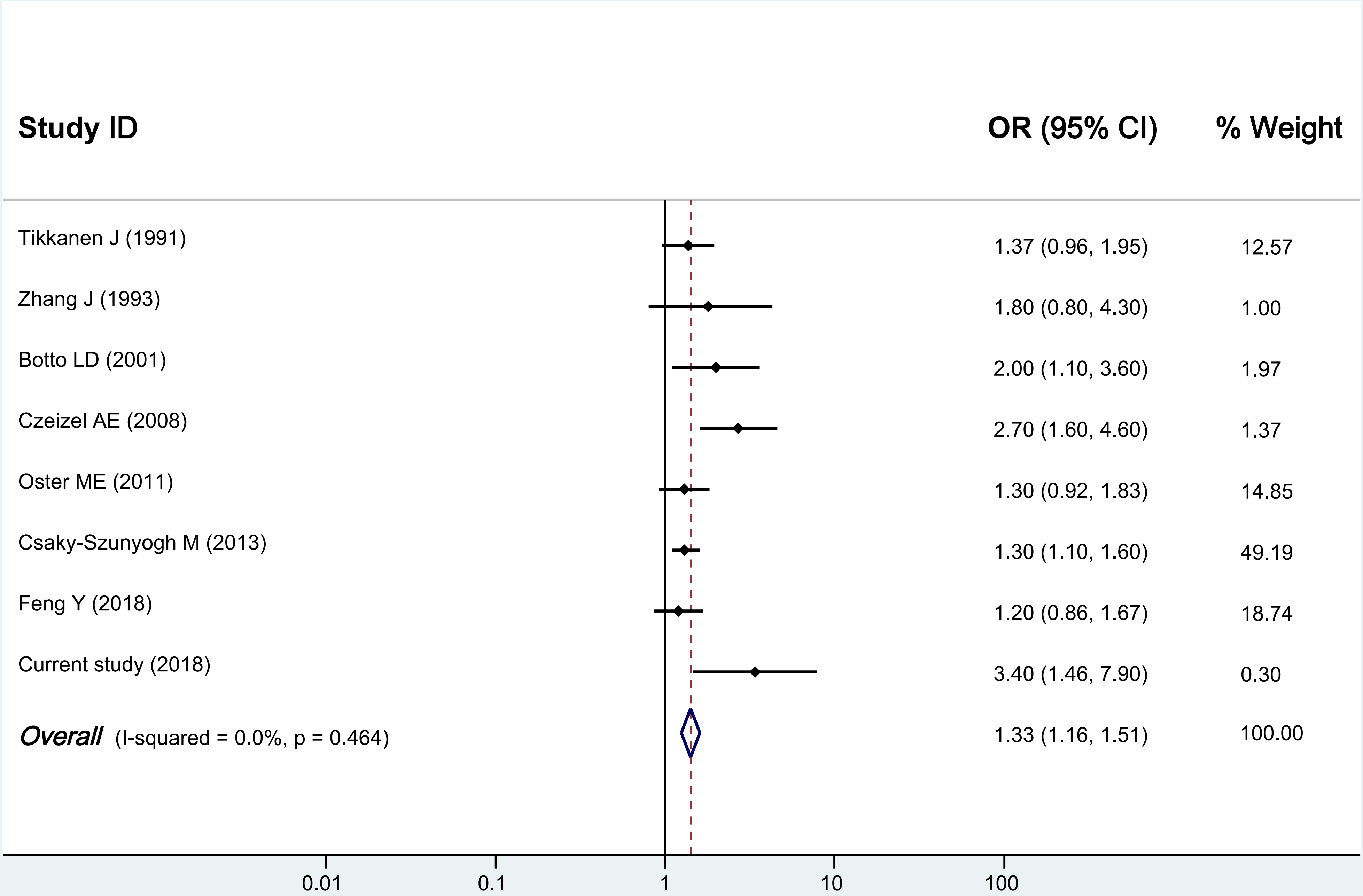

Figure S4 Forest plot of ORs and 95% CIs for the association of maternal upper respiratory tract infection/influenza and VSD--- after a propensity-score-matched analysis

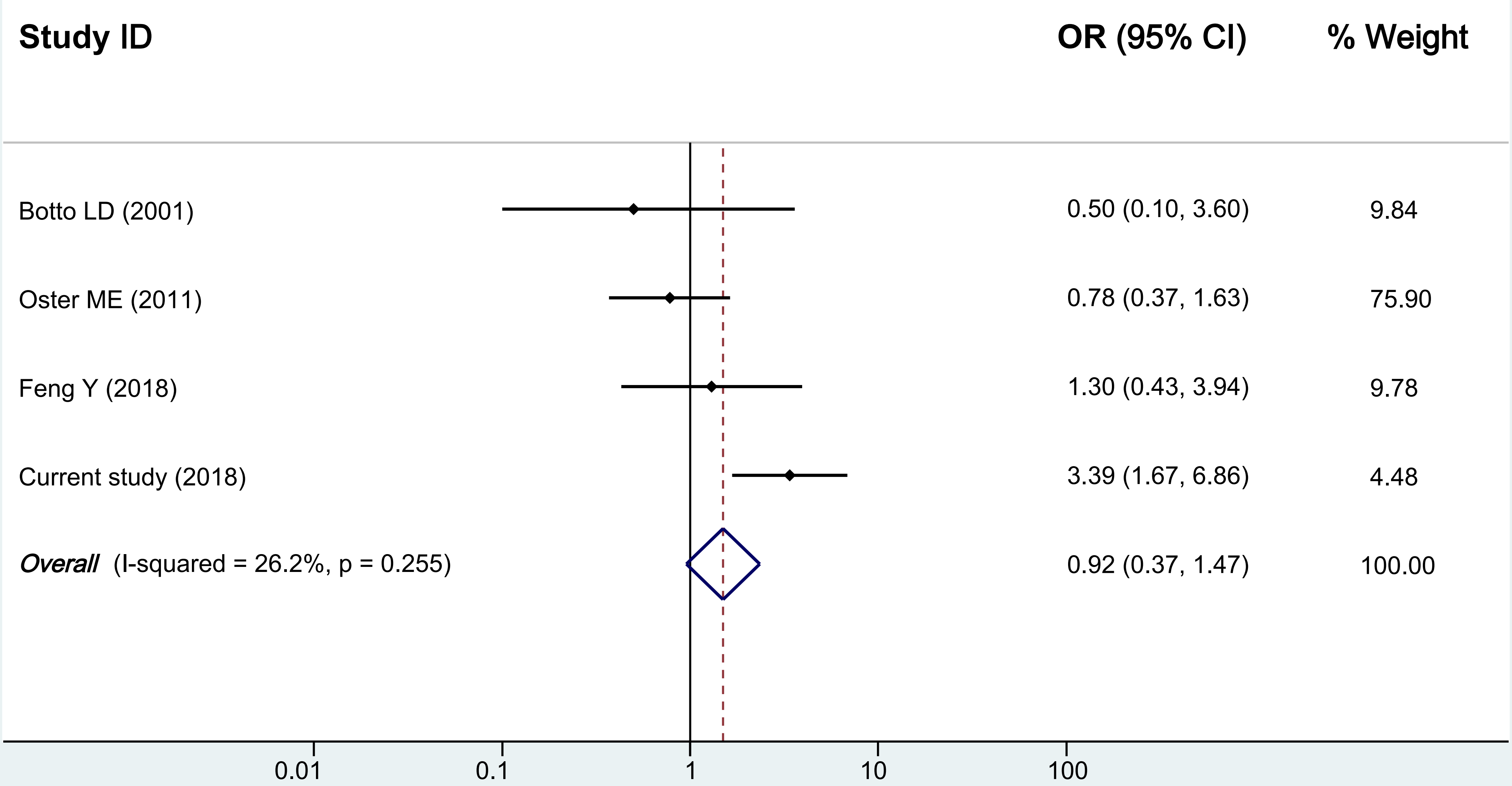

Figure S5 Forest plot of ORs and 95% CIs for the association of maternal upper respiratory tract infection/influenza and TOF--- after a propensity-score-matched analysis

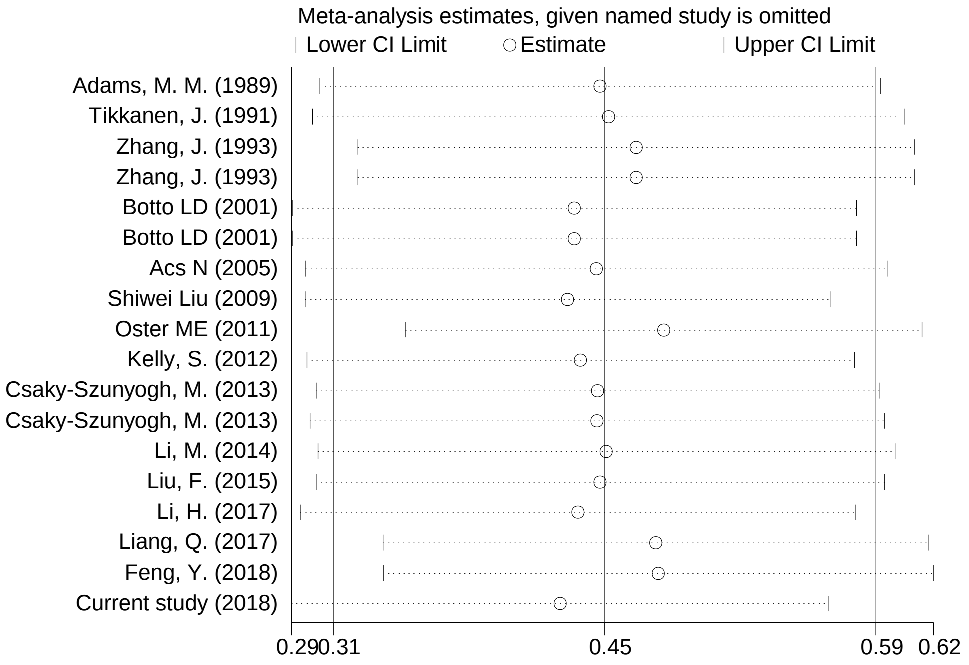

Figure S6 Plot of sensitivity analysis for the association of maternal upper respiratory tract infection/influenza and CHD

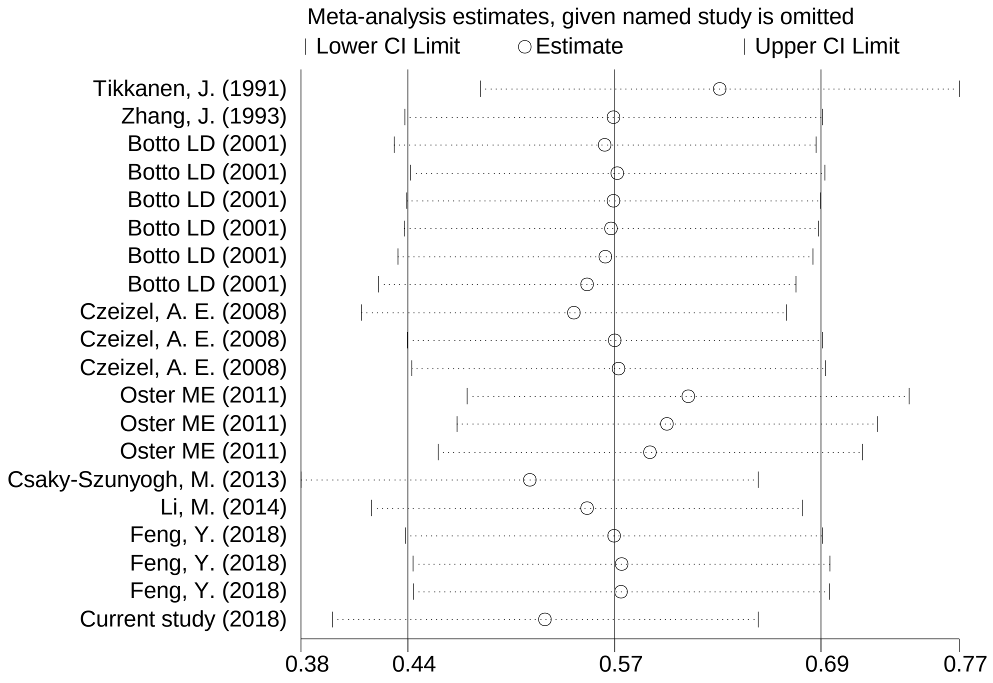

Figure S7 Plot of sensitivity analysis for the association of maternal upper respiratory tract infection/influenza and simple CHD

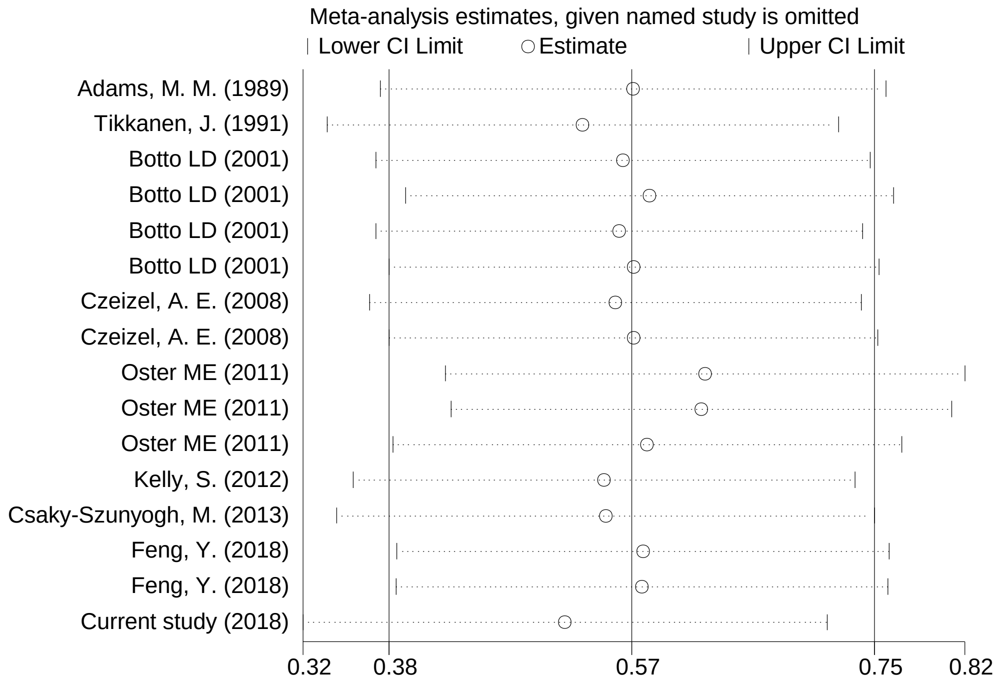

Figure S8 Plot of sensitivity analysis for the association of maternal upper respiratory tract infection/influenza and complex CHD

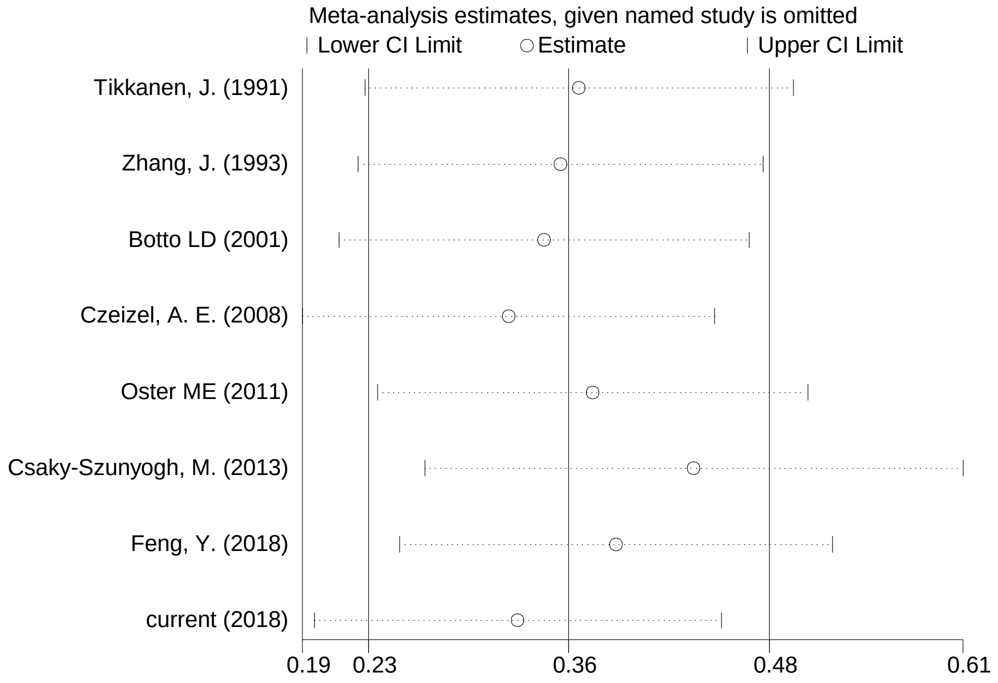

Figure S9 Plot of sensitivity analysis for the association of maternal upper respiratory tract infection/influenza and VSD

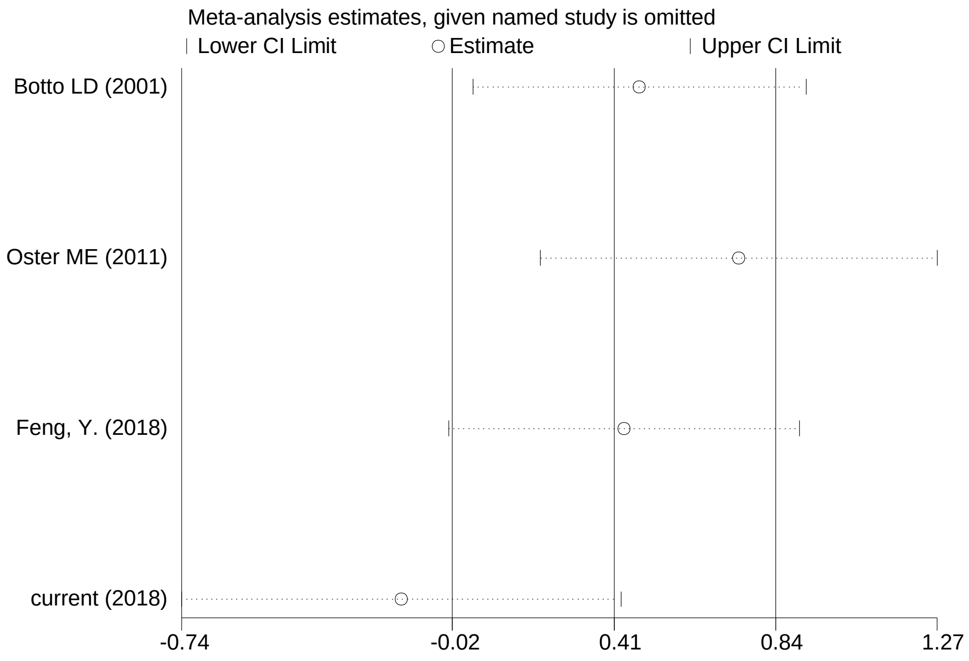

Figure S10 Plot of sensitivity analysis for the association of maternal upper respiratory tract infection/influenza and TOF
